# Relative Validity of a Food and Beverage Preference Questionnaire to Characterize Taste Phenotypes in Children Adolescents and Adults

**DOI:** 10.3390/nu11071453

**Published:** 2019-06-27

**Authors:** Hannah Jilani, Hermann Pohlabeln, Stefaan De Henauw, Gabriele Eiben, Monica Hunsberger, Dénes Molnar, Luis A. Moreno, Valeria Pala, Paola Russo, Antonia Solea, Toomas Veidebaum, Wolfgang Ahrens, Antje Hebestreit, on behalf of the IDEFICS and I.Family consortia

**Affiliations:** 1 Leibniz Institute for Prevention Research and Epidemiology—BIPS, 28359 Bremen, Germany; 2 Institute for Public Health and Nursing Science, University of Bremen, 28359 Bremen, Germany; 3 Department of Public Health, Ghent University, B-9000 Ghent, Belgium; 4 Department of Biomedicine and Public Health, School of Health and Education, University of Skövde, 54128 Skövde, Sweden; 5 Department of Public Health and Community Medicine, University of Gothenburg, 40530 Gothenburg, Sweden; 6 Department of Pediatrics, Medical School, University of Pécs, 7623 Pécs, Hungary; 7 GENUD (Growth, Exercise, Nutrition and Development) Research Group, Faculty of Health Sciences, University of Zaragoza, 50009 Zaragoza, Spain; 8 Department of Research, Epidemiology and Prevention Unit, Fondazione IRCCS Istituto Nazionale dei Tumori, 20133 Milan Italy; 9 Institute of Food Sciences, National Research Council, 83100 Avellino, Italy; 10 Research and Education Institute of Child Health, 2035 Strovolos, Cyprus; 11 Department of Chronic Diseases, National Institute for Health Development, 11619 Tallin, Estonia; 12 Institute of Statistics, Faculty of Mathematics and Computer Science, University of Bremen, 28359 Bremen, Germany

**Keywords:** taste preference questionnaire, validation, European children, adolescents, adults

## Abstract

To assess the relative validity of our food and beverage preference questionnaire we investigated the association between sweet and fatty taste preference scores (assessed using a food and beverage preference questionnaire) and sweet and fatty food propensity scores (derived from a food frequency questionnaire). In I.Family, a large European multi-country cohort study, 12,207 participants from Cyprus, Estonia, Germany, Hungary, Italy, Spain and Sweden, including 5291 adults, 3082 adolescents, and 3834 children, completed a food and beverage preference questionnaire with 63 items. Cumulative preference scores for sweet and fatty taste were calculated from the single item ranking ranging from 1 to 5. The relative consumption frequency of foods classified as sweet and fatty was used to calculate the corresponding consumption propensities, a continuous variable ranging from 0 to 100. We conducted regression analyses to investigate the association between sweet and fatty taste preference scores and sweet and fatty food propensity scores, respectively, separately for adults, adolescents ≥12 years, and for children <12 years. The overall sweet taste preference score was positively associated with the sweet food consumption propensity score (β = 2.4, 95% CI: 2.1;2.7) and the fatty taste preference score was positively associated with the fatty food consumption propensity score (β = 2.0, 95% CI: 1.8;2.2). After stratification for age (children <12 years, adolescents ≥12 years, and adults), the effect remained significant in all age groups and was strongest in adolescents and adults. We conclude that our food and beverage preference questionnaire is a useful instrument for epidemiological studies on sensory perception and health outcomes and for the characterization of sensory taste phenotypes.

## 1. Introduction

Methods to accurately measure sensory taste perception are often laborious. A high degree of standardization of the assessment method is a prerequisite for valid results. In large multi-country epidemiological studies it is often not feasible to conduct sensory perception tests according to the standards that can only be reached in a laboratory setting. As an alternative, taste preference questionnaires have previously been applied to investigate the association between taste preferences and health outcomes [1,2,3]. Only one questionnaire, originally developed for French adults, has so far been developed for a large epidemiological study [4]. While the first part of the questionnaire assesses standard taste preferences, it also assesses the liking of seasoning and whole dishes, for example, in later parts. As this questionnaire is quite elaborate and includes many items that are specific to France, it might not be suitable for children and adolescents in general and also not for use throughout Europe. To date, no study has applied a food and beverage preference questionnaire in both children and their parents at the same time.

The purpose of this study was to analyze the validity of data obtained using a food and beverage preference questionnaire (FBPQ) developed in the context of I.Family, a large pan-European study comprising children, adolescents, and adults. Determining the validity of the questionnaire will help further the investigation of the association between taste preferences and health outcomes. To this end we developed a FBPQ for children, adolescents, and adults and applied it in I.Family. As a measure of the relative validity of the questionnaire, we analyzed the association between self-reported sweet and fatty taste preferences assessed by the FBPQ and self-reported food consumption frequencies of sweet and fatty foods assessed by a food frequency questionnaire (FFQ).

## 2. Materials and Methods

I.Family is a large multi-country longitudinal cohort study and the follow-up study of the IDEFICS (Identification and prevention of Dietary- and lifestyle-induced health Effects in Children and infantS) study [5,6]. Between March 2013 and April 2014, all children that participated in the IDEFICS study were invited to a follow-up examination together with their siblings and parents. In the sensory taste perception module, we included all participants aged six years and above. This resulted in a study sample of 12,207 participants from Cyprus, Estonia, Germany, Hungary, Italy, Spain, and Sweden who completed the food and beverage preference questionnaire and provided all co-variables.

Each study center obtained ethical approval from its local institutional review board (e.g., Cyprus National Bioethics Committee, Nicosia, Cyprus; Tallinn Medical Research Ethics Committee, Tallinn, Estonia; Ethics Committee of the University of Bremen, Bremen, Germany; Egészségügyi Tudományos Tanács, Pécs, Hungary; Azienda Sanitaria Locale Avellino Comitato Etico, Avellino, Italy; Regionala Etikprövningsnämnden i Göteborg, Gothenburg, Sweden; Comité Ético de Investigación Clínica de Aragón, Zaragoza, Spain). Parents gave written informed consent for themselves and for their young children (up to 11 years). Adolescents 12 years and older gave their own written informed consent. All children were informed orally and gave their oral consent to participate in the study.

### 2.1. Food and Beverage Preference Questionnaire

Based on two existing tools, we developed an FBPQ suitable for assessment in children from the age of six years. One of the tools was administered to children and tested for reliability [7] and the other was administered to adults and internally validated [4]. A detailed description of the FBPQ has been published elsewhere [8]. The questionnaire consisted of 63 pictures of single foods (e.g., banana, spinach), mixed foods (e.g., hot dog, kebab), condiments (e.g., jam, mayonnaise) and drinks (e.g., coke, lemonade). A pre-test was conducted in every country to ensure the feasibility of all food items across countries and to find out how long participants would require to complete the questionnaire. The estimated time for the completion of the food and beverage preference questionnaire was 7 min. Each subject ranked his/her own preference for the taste of the respective food or drink on a 5-point Likert (smiley-) scale, with 1 meaning “do not like at all” and 5 meaning “like very much”. Subjects could indicate if they did not know or had never tasted a given food item.

#### Sensory Taste Preference Score

Cumulative preference scores for sweet, fatty, salty, and bitter tastes were calculated from the single item rankings, as described in a previous publication [8]. First, we excluded foods that were rated by less than 75% of the participants. Further, data of participants with more than 20 missing or “Never tried/Don’t know” answers were excluded. In principle, “Never tried/Don’t know” responses were set to “missing”. A latent variable exploratory factor analysis was then conducted [9] to assess the associations between foods and beverages. The age- and sex-specific factor analysis was conducted for the strata boys <12 years, girls <12 years, boys ≥12 years, girls ≥12 years, and adults ≥18 years. A food or drink item was considered to belong to a particular factor if the factor loading was greater than 0.30 on the specific factor. Thereafter, a content analysis was conducted to assign the factors to the taste modalities sweet, salty, fatty, and bitter (Table 1). Food and drink items with no load on one or more of the factors were not included in further analyses.

Taste preference scores were calculated separately for each stratum, based on the mean liking of the foods and drinks assigned to each of the 4 taste modalities. To this end, the sum of the ratings for the foods and drinks was calculated and divided by the number of foods and drinks that were included in the specific taste modality group. In the present analysis, only the sweet and fatty taste preference scores were considered.

### 2.2. Food Frequency Questionnaire

In I.Family, each participant completed the adapted version of the validated [10,11] and reproducibility tested [12] FFQ used in the IDEFICS study. Parents completed the FFQ for children below the age of 12 years as it has been shown that they might be unreliable reporters of their diet [13]. Parents and adolescents 12 years and older reported on their own diet. The FFQ contained 59 items including 19 fatty items (fried potatoes, whole fat milk, whole fat yoghurt, fried fish, cold cuts/sausages, fried meat, fried poultry, fried eggs, mayonnaise and mayonnaise based products, cheese, chocolate- or nut-based spread, butter/margarine on bread, oil, nuts and seeds, salty snacks, savoury pastries, chocolate-based candies, cake/pudding/cookies, and ice cream) and 16 sweet items (fresh fruit with added sugar, fruit juices, carbonated sugar sweetened drinks, sugar sweetened drinks not carbonated, sweetened coffee, sweetened tea, sweetened or sugar added breakfast cereals, sweetened and/or flavored milk, sweetened and/or flavored yoghurt, jam, honey, chocolate or nut based spreads, chocolate-based candies, non-fat candies, cake/pudding/cookies, and ice cream). The response categories were “1–3 times a week”, “4–6 times a week”, “1 time/day”, “2 times a day”, “3 times a day” and “Never/less than once a week”. The obtained answers were converted into weekly consumption frequency, ranging from 0 to 28. The weekly consumption frequencies of named fatty foods, sweet foods, and of all foods included in the FFQ were summed up. The sweet and fatty food propensity scores in terms of the relative consumption frequency of named sweet or fatty foods were calculated. This was done by dividing the consumption frequency of the sweet and fatty foods by the consumption frequency of all foods included in the FFQ multiplied by 100. This resulted in a continuous variable, indicating the sweet and fatty food propensity scores ranging from 0% to 100% [14,15]. Thus, the scores reflect the proportion of sweet and fatty foods in the whole diet. For example, a sweet propensity score of 25 indicates that 1/4 of all food items consumed in one week were foods high in sugar. As the ultimate aim of the IDEFICS Study and I.Family was to investigate risk factors for child and adolescent health, such as obesity and to describe the obesogenic food environment and healthy or unhealthy food choices, the FFQ was designed to measure consumption frequency of obesogenic foods, which primarily contain sugar and fat. Hence, the FFQ was designed to allow the expression of the consumption frequency of sweet and fatty foods rather than of bitter and salty foods and may be used to describe the tendency to choose sweet or fatty food alternatives over foods lower in sugar or fat. Whenever the FFQ contained a sweetened and an unsweetened alternative of a food group (e.g., sweetened vs. unsweetened milk products or sweetened fruits vs. unsweetened fresh fruits), the sweetened alternative was included in the food consumption propensity score. This was done to describe the behavior to choose the sweetened alternative over the unsweetened alternative.

### 2.3. Questionnaires and Anthropometry

Self-completion questionnaires were used to assess age, sex, country of residence, and highest level of education. For each parent we categorized the highest educational level acquired according to the International Standard Classification of Education (ISCED), ranging from 1 (low education) to 8 (high education) [16]. For the present analysis, the education level was grouped into three categories; “low education” (ISCED level 0–2), “medium education” (ISCED level 3–5) and “high education” (ISCED level 6–8). The family affiliation was assessed using a kinship interview. Parents completed questionnaires for themselves as well as for their children under the age of 12 years. Adolescents 12 years and older completed the questionnaire on their own.

The height and weight of all participants were measured in a fasting state. The body mass index (BMI) was calculated for all participants and converted into age-and sex-specific z-scores for all children and adolescents [17]. For adults, the cut off of 25 kg/m^2^ was chosen to classify parents as overweight, including obese [18].

### 2.4. Statistical Analysis

We calculated the characteristics of the study sample separately for adults, adolescents ≥12 years, and for children < 2 years. For further descriptive analysis we categorized the sweet and fatty taste preference score into 4 categories separately for sweet and fatty. In category 1 we assigned all taste preference scores ranging from 1 to <2, in category 2 all taste preference scores ranging from 2 to <3, in category 3 all taste preference scores ranging from 3 to <4 and in category 4 all taste preference scores ranging from 4 to 5. After this, we calculated the mean, standard deviation, and lower and upper quartiles (p25, p75) for the sweet food propensity score (stratified by sweet taste preference score categories), as well as for the mean fatty food propensity score (stratified by fatty taste preference score categories) separately for adults, adolescents ≥12 years, and for children <12 years. We conducted regression analyses to investigate the association between sweet and fatty taste preference scores and sweet and fatty food propensity scores, respectively, separately for adults, adolescents ≥12 years, and for children <12 years. This we did separately for adults, adolescents ≥12 years, and for children <12 years. In the regression model we included the sweet and fatty taste preference scores as non-categorized independent variables, whereas the sweet and fatty food propensity scores were considered as the dependent variables. We adjusted all models for sex, age, BMI (for children and adolescents: BMI z-score), highest education level, and country of residence as fixed factors and family affiliation as a random factor. As associations between weight status and food intake have been described in previous studies [19,20], it is important to conduct stratified analyses. Thus, we investigated data not only in the full sample (including all individuals), but also separately for underweight and overweight/obese participants. Further stratified analyses were performed according to sex, country, and education level.

All regression analyses were carried out using PROC MIXED (SAS version 9.3). Effect estimates were presented with the corresponding 95% confidence intervals (95% CI) and *p*-values.

## 3. Results

The study sample consisted of 5291 adults, 3082 adolescents, and 3834 children. The mean age of the total sample was 24.8 years and 40% were overweight or obese. The highest proportion of participants was from Cyprus (21.5%) and the smallest from Spain (6.7%). The mean sweet and fatty taste preference scores for the total sample were 3.8 for each of the tastes. The mean sweet food propensity score was 21.3 and the mean fatty food propensity score was 24.2. More detailed characteristics can be found in Table 2.

In Table 3 the mean sweet and fatty food propensity scores within each sweet and fatty taste preference category are displayed separately for adults, adolescents ≥12 years, and for children <12 years. The results show that the consumption of sweet and fatty foods generally increased as the sweet and fatty taste preference scores increased, respectively. It is only in children that the mean sweet and fatty food propensity scores in the lowest sweet and fatty preference score categories were higher than in the other sweet and fatty preference score categories, respectively.

Table 3 shows the effect estimates of the association between sweet and fatty taste preference scores and sweet and fatty food propensity scores, respectively, separately for adults, adolescents ≥12 years and for children <12 years. A positive association could be seen for sweet and fatty taste in adults (sweet: β = 3.1, 95% CI: 2.7; 3.5, fatty: β = 2.3, 95% CI: 2.0; 2.6) and adolescents (sweet: β = 3.0, 95% CI: 2.3; 3.6, fatty: β = 2.9, 95% CI: 2.4; 3.4). The association in children was weaker but still positive (sweet: 0.8, 95% CI: 0.3; 1.2, fatty: β = 0.5, 95CI: 0.1; 0.9). In the overall sample, the sweet taste preference score was positively associated with the sweet food propensity score (β = 2.4, 95% CI: 2.1; 2.7) and the fatty taste preference score was positively associated with the fatty food propensity score (β = 2.0, 95% CI: 1.8; 2.2). Further, the Table S1 (see supplement) shows the effect of all included co-variables on the sweet and fatty food consumption frequencies.

Stratified analyses by country, sex, weight status, and education level showed that the association between taste preference scores and food propensity scores remained stable within the strata (Table 4). A few differences could be observed, however. When stratifying the regression analyses by sex, no differences were observed between female and male adolescents and adults. Among children, on the other hand, the association was stronger in girls than in boys. When stratifying the regression analyses by country, the associations were again present and positive in all countries for adolescents and adults. For children, the associations were not positive for sweet in Sweden and Hungary and for fatty in Italy, Estonia, and Sweden. When stratifying by weight status and education level, the same patterns as in the full sample could be seen. The association was present and positive in adolescents and adults. While the association was still positive for children, for the fatty taste it was no longer significant for overweight children and children with parents with low/medium or high education.

## 4. Discussion

Self-reported sweet and fatty taste preferences were positively associated with self-reported sweet and fatty food propensity scores, respectively, in children, adolescents, and adults. This indicates that taste preferences are indeed associated with actual food choices; the higher the sweet or fatty preference score, the higher the sweet or fatty propensity score, respectively. Overall the consumption of sweet and fatty foods increased by 2% per unit increase of the sweet and fatty preference score category. For adolescents and parents, the increase was 3% and for children it was between 0.5% and 1% per unit increase. The strength of the association was strongest for adults and adolescents. The estimates for children 6 to 12 years old were weaker, suggesting that younger children consume food and drink offered at home by their parents. As parents act as gatekeepers with regard to the availability of food and drink [21] children might not be able to consume only what their taste preferences would imply but rather what their parents want them to consume. This might have attenuated the investigated association in the group of children. Our FBPQ may thus be considered as a useful instrument to provide valid data on self-reported sweet and fatty taste preferences for multi-country epidemiological field studies.

The questionnaire used in I.Family to assess taste preference in children, adolescents, and their parents was developed on the basis of two existing tools. As no questionnaire for taste preference assessment in children, adolescents, and adults across Europe existed, there was a need to develop and test the validity of the I.Family FBPQ. Two recent studies validated their food and beverage preference questionnaires to administer to adults across two cultures; English and Arab [22] and Australian and Thai [23]. Besides questionnaires for observation studies, food and beverage preference questionnaires were tested for reliability and validity both in a laboratory setting [24] and under free-living conditions [24,25,26]. Thus, validated preference questionnaires exist, but the present study contributes a validated questionnaire allowing for investigating associations between sensory taste preferences and health outcomes, even in a cross-cultural setting including a wide range of age-groups.

As we had no objective measurement to validate the FBPQ, we used the self-reported food consumption frequencies for the validation. The validity of the FFQ itself could be questioned due to social desirability or recall bias and misclassification may potentially attenuate the observed associations. While we acknowledge the possible attenuation, we nevertheless believe that our FFQ provides useful information since it has been previously validated [10,11] and tested for reproducibility [12].

In comparison to the FFQ, the FBPQ is a tool especially designed to assess taste preferences. It has been analyzed before and has been found to be applicable in children from the age of six years upward [8]. This instrument is easy to apply and faster to complete compared to an FFQ. Further, as it is not necessary to recall the diet of the previous month, it is likely to provide more robust information with regard to recall bias.

The confirmation of the relative validity of the FBPQ is important as the tool can be used in future studies to investigate additional aspects of taste preferences, such as the longitudinal development of taste preferences and possible associations with health outcomes in young European populations. With respect to so-called “upstream factors” of taste preference development, studies investigating regional or temporal changes of determinants will also be able to make use of the present results. Further, the associations observed in this study will contribute toward the development of successful interventions, health programs, and policies aiming at improving the dietary behavior of children as well as adolescents and adults.

### Limitations and Strengths

Our study has some limitations that need to be addressed. The FFQ for children below the age of 12 years was proxy-reported by the parents, hence social desirability potentially affected our data. Parents might have responded to the questions of the FFQ in a way they thought to be more socially acceptable. This might have led to an attenuation of the studied association. In addition, as the number of children in the lowest sweet and fatty taste preference score category was very small, the calculation of sweet and fatty food propensity scores within those categories was based on very small numbers and was thus not representative for this age-group. Despite this potential limitation, we are nevertheless convinced that the results presented in the current paper provide important information for public health stakeholders, policy makers, and researchers.

In the present study, information on restrained eating could not be considered. This could possibly have led to an attenuation of our results regarding the association between taste preferences and food consumption frequency. We obtained information on current dieting only for adolescents. In a sensitivity analysis within the group of adolescents, we adjusted the regression analysis for currently being on a diet and could see that the associations under investigation remained positive and significant. Unfortunately, we could not adjust the whole analysis for restrained eating, but the results of the sensitivity analysis suggest that this does not alter the investigated association.

Another limitation concerns the unequal distribution of the sexes within the group of adults, whereby the majority was female. The stratified results, however, showed no differences between men and women.

The strengths of our study are the large multi-country study sample and the broad age-range of participants. Due to the large study sample and the assessment of a broad range of health-related information, we were able to adjust the analysis for several covariates, such as sex, weight status, country, and education level. Further, we were even able to also include data of underweight and overweight/obese participants. In order to account for the bidirectional association between weight status and food consumption/energy intake, we analyzed the data stratified by weight status. The studied associations were positive and significant for under-/normal weight as well as for overweight/obese participants. The finding, which was that the association for fatty taste was not significant in children, could be due to the fact that parents of overweight/obese children are more restrictive with regard to sweet and fatty foods. This would then lead to the observed attenuation of the association between taste preferences and food consumption frequencies.

## 5. Conclusions

Although food choices are influenced by various factors, we were able to show a positive association between sweet and fatty taste preferences, assessed via an FBPQ, and sweet and fatty food propensity scores, assessed via a validated FFQ, in a large multi-country epidemiological cohort study in children, adolescents, and adults. We conclude that our FBPQ is a valid instrument for epidemiological field studies aiming to characterize taste phenotypes.

## Figures and Tables

**Table 1:** Food and drinks representing the four taste modalities [8].

	Boys <12 Years	Girls <12 Years	Boys ≥12 Years	Girls ≥12 Years	Fathers	Mothers
**Sweet**						
Milk chocolate	X	X	X	X	X	X
Chocolate bar	X	X	X	X	X	X
Lemonade	X	X		X	X	X
Coke	X	X	X	X	X	X
Diet coke	X		X	X	X	X
Donut	X		X	X	X	X
Jam	X	X	X	X		X
Honey	X	X	X	X	X	X
Plain croissant		X	X	X	X	X
Chocolate croissant	X	X		X	X	X
Cornflakes	X	X	X	X	X	X
Chocolate crispies		X	X	X	X	X
Chocolate spread	X	X	X	X	X	X
Banana	X	X		X	X	
Fruit yoghurt	X	X	X	X	X	X
Yoghurt	X		X	X	X	X
Fruit juice	X	X	X	X	X	X
Chocolate pudding		X	X			
Gateau			X		X	X
Ice tea					X	
Ice cream					X	X
Water					X	
Wholemeal bread					X	
**Salty**						
Salt			X			
Salted nuts	X	X	X	X	X	X
Salted pistachios	X	X	X	X	X	X
Savoury biscuits	X	X	X	X	X	X
Salty sticks	X	x	X	X	X	X
Olives					X	
Feta					X	
**Fatty**						
Hamburger	X	X	X	X	X	X
Hot Dog	X	X	X	X	X	X
Fried chicken	X		X	X	X	X
Steak	X			X	X	
French fries	X	X	X	X		X
Chips	X	X	X	X		X
Sausage	X	X	X	X	X	X
Salami	X			X	X	X
Butter	X	X	X	X	X	X
Mayonnaise	X		X	X	X	X
Milk		X		X	X	
Cream			X	X	X	X
Mashed potatoes			X			
Kebab				X	X	X
Nachos				X		X
Chili sauce				X		X
**Bitter**						
Broccoli	X	X	X	X	X	X
Spinach	X	X	X	X	X	X
Lettuce	X		X			
Olives	X		X			X
Lasagne			X			
Red cabbage					X	X
Sprouts					X	X
Asparagus					X	X
Grapefruit						X
Steak						X

**Table 2:** Characteristics of the study sample.

	Adults *n* = 5291	Adolescents *n* = 3082	Children *n* = 3834	Total *n* = 12,207
	Mean (SD) (*p*25; *p*75)	Mean (SD) (*p*25; *p*75)	Mean (SD) (*p*25; *p*75)	Mean (SD) (*p*25; *p*75)
Age	42.4 (5.8) (38.4; 46.2)	13.6 (1.0) (12.8; 14.0)	9.6 (1.6) (8.8; 10.8)	24.8 (15.9) (11.0; 41.0)
Sweet food propensity score	18.3 (11.5) (9.4; 25.5)	24.5 (11.1) (16.5; 31.1)	22.9 (10.3) (15.7; 29.0)	21.3 (11.3) (13.0; 28.4)
Fatty food propensity score	22.0 (9.1) (15.6; 28.2)	24.5 (9.1) (18.3; 30.1)	27.0 (8.9) (21.2; 32.5)	24.2 (9.3) (17.8; 30.2)
Sweet preference score	3.5 (0.7) (3.0; 4.0)	4.0 (0.6) (3.6; 4.4)	4.1 (0.6) (3.4; 4.6)	3.8 (0.7) (3.3; 4.4)
Fatty preference score	3.5 (0.8) (3.0; 4.0)	4.0 (0.6) (3.6; 4.4)	4.1 (0.6) (3.8; 4.6)	3.8 (0.7) (3.3; 4.4)
	**N (%)**	**N (%)**	**N (%)**	**N (%)**
Female	3490 (66.0)	1584 (51.4)	1896 (49.5)	7242 (57.0)
Overweight/obese	2988 (56.5)	857 (27.8)	1046 (27.3)	4891 (40.1)
All countries				
Cyprus	1151 (21.8)	691 (22.4)	781 (20.4)	2623 (21.5)
Estonia	761 (14.4)	478 (15.5)	580 (15.1)	1819 (14.9)
Germany	834 (15.8)	466 (15.1)	550 (14.4)	1850 (15.2)
Hungary	988 (18.7)	434 (14.1)	512 (13.4)	1934 (15.8)
Italy	720 (13.6)	589 (19.1)	682 (17.8)	1991 (16.3)
Spain	346 (6.5)	168 (5.5)	306 (8.0)	820 (6.7)
Sweden	491 (9.3)	256 (8.3)	423 (11.0)	1170 (9.6)

Abbreviations: *n* = number, SD = standard deviation, *p* = percentile.

**Table 3:** Mean sweet and fatty food propensity scores within sweet and fatty taste preference score groups and β estimates for the association between sweet and fatty taste preference scores and sweet and fatty food propensity scores.

	Adults	Adolescents	Children	Total
**Sweet preference score category (range)**	**Sweet propensity score (mean (SD)) (*n*)**			
1 (1–<2)	13.7 (12.1) (109)	17.7 (5.7) (6)	23.6 (11.9) (12)	14.8 (12.2) (127)
2 (2–<3)	15.3 (10.7) (1108)	21.2 (11.3) (181)	20.3 (9.7) (188)	16.6 (10.9) (1477)
3 (3–<-4)	18.3 (11.3) (2785)	23.1 (10.9) (1167)	22.3 (10.1) (1169)	20.3 (11.1) (5121)
4 (4–≤5)	21.1 (11.8) (1289)	25.8 (11.0) (1728)	23.5 (10.4) (2464)	23.6 (11.0) (5481)
β (95% CI) ^1,2^ *p*-value	3.1 (2.7;3.5) *p* < 0.0001	3.0 (2.3;3.6) *p* < 0.0001	0.8 (0.3;1.2) *p* = 0.001	2.4 (2.1;2.7) *p* < 0.0001
**Fatty preference score category (range)**	**Fat propensity score (mean (SD)) (*n*)**			
1 (1–<2)	15.7 (8.0) (185)	16.8 (11.8) (6)	27.7 (10.2) (14)	16.5 (8.8) (205)
2 (2–<3)	19.7 (8.8) (1066)	20.1 (9.3) (183)	26.0 (9.2) (168)	20.5 (9.1) (1417)
3 (3–<4)	22.3 (8.9) (2553)	23.0 (8.8) (1156)	26.7 (9.1) (1087)	23.5 (9.1) (4796)
4 (4–≤5)	23.8 (9.1) (1487)	26.1 (8.9) (1737)	27.2 (8.7) (2565)	26.0 (9.0) (5789)
β (95% CI) ^1,2^ *p*-value	2.3 (2.0;2.6) *p* < 0.0001	2.9 (2.4;3.4) *p* < 0.0001	0.5 (0.1;0.9) *p* = 0.02	2.0 (1.8;2.2) *p* < 0.0001

CI: Confidence interval; ^1^: Sweet and fatty preference scores entered the regression models as continuous variable; ^2^: Regression models were adjusted for sex, age, BMI, highest education level, and country of residence as fixed factors and family affiliation as random factor.

**Table 4:** Stratified results of the association between sweet and fatty taste preference and sweet and fatty food propensity scores (β estimates and 95% CI).

	Adults	Adolescents	Children
	**Sweet food consumption score β (95% CI)**		
**Male and Female**	3.1 (2.7;3.5)	3.0 (2.3;3.6)	0.8 (0.3;1.2)
**Male**	2.8 (2.0; 3.6)	2.7 (1.8; 3.6)	0.4 (−0.1; 1.3)
**Female**	3.1 (2.6; 3.6)	3.2 (2.3; 4.2)	0.8 (0.2; 1.5)
**Under-/normal weight**	3.2 (2.6; 3.9)	2.7 (1.9; 3.5)	0.6 (0.1; 1.2)
**Overweight/Obese**	2.9 (2.3; 3.4)	3.4 (2.2; 4.7)	1.0 (0.01; 2.0)
**Low/medium** **education level**	2.9 (2.3; 3.5)	3.2 (2.3; 4.2)	1.0 (0.3; 1.7)
**High** **education level**	3.1 (2.6; 3.7)	2.6 (1.7; 3.6)	0.6 (−0.0; 1.2)
	**Fatty food consumption score β (95% CI)**		
**Male and Female**	2.3 (2.0;2.6)	2.9 (2.4;3.4)	0.5 (0.1;0.9)
**Male**	2.2 (1.7; 2.8)	2.4 (1.6; 3.2)	0.4 (−0.3; 1.0)
**Female**	2.2 (1.8; 2.5)	3.2 (2.6; 4.0)	0.7 (0.1; 1.2)
**Under-/normal weight**	2.6 (2.1; 3.0)	3.0 (2.3; 3.6)	0.7 (0.2; 1.1)
**Overweight/Obese**	1.9 (1.5; 2.3)	2.8 (1.8; 3.9)	0.4 (−0.5; 1.3)
**Low/medium** **education level**	2.3 (1.9; 2.8)	2.8 (2.0; 3.5)	0.4 (−0.2; 1.1)
**High** **education level**	2.1 (1.6; 2.5)	3.1 (2.4; 3.9)	0.5 (−0.0; 1.0)

## Supplementary Materials

The following are available online at https://www.mdpi.com/2072-6643/11/7/1453/s1, Table S1: Estimates of fixed effect sizes with reference category in parentheses in relation to sweet and fatty food consumption frequencies in adults, adolescents and children.
