# Supplementary material for: Relative Validity of a Food and Beverage Preference Questionnaire to Characterize Taste Phenotypes in Children Adolescents and Adults
Source: Nutrients. 2019 Jun 27;11(7):1453. doi: 10.3390/nu11071453 (PMC6682919; doi:10.3390/nu11071453)
Supplement: Supplementary file 1 [file nutrients-11-01453-s001.pdf]

Table S1: Estimates of fixed effect sizes with reference category in parentheses in relation to sweet and fatty food consumption frequencies in adults, adolescents and children.

Food consumption frequency	Covariate (Ref)		Adults β (CI)	Adults p	Adolescents β (CI)	Adolescents p	Children β (CI)	Children p
Sweet	Sex (Female)	Male	1.8 (1.2; 2.3)	<0.0001	1.0 (0.3; 1.8)	0.007	0.8 (0.3; 1.4)	0.003
	Country (Spain)	Italy	0.3 (-1.2; 1.8)	0.7	-0.07 (-1.9; 1.9)	1.0	1.8 (0.4; 3.2)	0.01
		Estonia	0.1 (-1.4; 1.5)	0.9	1.2 (-0.6; 3.1)	0.2	2.8 (1.4; 4.1)	< 0.0001
		Cyprus	-2.6 (-4.0; -1.2)	0.0002	-1.2 (-3.0;0.6)	0.2	1.6 (0.26; 2.9	0.02
		Sweden	-6.8 (-8.4; 5.3)	<0.0001	-4.7 (-6.8; -2.7)	<0.0001	-4.4 (-5.8; -2.9)	< 0.0001
		Germany	4.4 (2.9; 5.8)	<0.0001	4.0 (2.1; 5.8)	<0.0001	7.7 (6.3; 9.2)	< 0.0001
		Hungary	4.0 (2.6; 5.4)	<0.0001	0.6 (-1.3; 2.6)	0.5	4.3 (2.9; 5.7)	< 0.0001
	BMI z-score		-0.11 (-0.2; -0.1)	<0.0001	-0.3 (-0.4; -0.2)	<0.0001	-0.3 (-0.4; -0.2)	< 0.0001
	ISCED (Low)	Medium	-1.3 (-2.9; 0.4)	0.1	-2.9 (-4.6; -1.3)	0.0006	-3.7 (-5.4; -2.1)	< 0.0001
		High	-3.5 (-5.2; -1.8)	0.3605	-5.4 (-7.2; -3.7)	<0.0001	-5.4 (-7.1; -3.7)	< 0.0001
Fat	Sex (Female)	Male	1.2 (0.7; 1.6)	<0.0001	1.0 (0.4; 1.6)	0.002	0.3 (-0.2; 0.8)	0.2
	Country (Spain)	Italy	-3.8 (-5.0; -2.7)	<0.0001	0.7 (-0.9; 2.2)	0.4	-3.5 (-4.7; -2.2)	< 0.0001
		Estonia	2.9 (1.8; 4.0)	<0.0001	0.3 (-1.3; 1.8)	0.7	0.8 (-0.4; 2.1)	0.2
		Cyprus	-5.4 (-6.5; -4.3)	<0.0001	-1.7 (-3.2; -0.3)	0.02	-3.0 (-4.2; -1.8)	< 0.0001
		Sweden	-1.6 (-2.8; -0.4)	0.01	-2.7 (-4.4; -1.0)	0.002	-4.0 (-5.4; -2.7)	< 0.0001
		Germany	3.1 (2.0; 4.2)	<0.0001	1.9 (0.3; 3.4)	0.02	1.2 (-0.0; 2.5)	0.06
		Hungary	-0.9 (-2.0; 0.2)	0.1	0.1 (-1.4; 1.7)	0.9	-4.0 (-5.3; -2.7)	< 0.0001
	BMI z-score		-0.2 (-0.2; -0.1)	<0.0001	-0.3 (-0.4; -0.3)	<0.0001	-0.2 (-0.3; -0.1)	< 0.0001
	ISCED (Low)	Medium	0.1 (-1.2; 1.4)	0.9	-1.1 (-2.4; 0.3)	0.1	-0.8 (-2.2; 0.7)	0.3
		High	0.2 (-1.1; 1.5)	0.8	-1.7 (-3.1; -0.3)	0.02	-0.8 (-2.3; 0.7)	0.3
